# Can a Short-Term Intervention Promote Growth Among Parents of Children with ASD?

**DOI:** 10.1007/s10803-025-06744-9

**Published:** 2025-02-21

**Authors:** Nagham Baransi, Miri Scharf

**Affiliations:** School of Therapy, Counseling and Human Development, University of Haifa, 199 Aba Khoushy Ave, Mount Carmel, 3498838 Haifa, Israel

**Keywords:** ASD, Mindset, Stress-related growth, Stress, Short-term intervention

## Abstract

The present study investigated the effects of a short-term synergic growth mindset intervention towards abilities and towards stress on reducing parental stress and promoting stress-related growth (SRG) among Arab parents of children diagnosed with Autistic Spectrum Disorder (ASD). One hundred and seven parents (70 mothers, 37 fathers) of male children with ASD, completed several questionnaires, including a demographic questionnaire; the Childhood Autism Rating Scale, the Parenting Stress Index-Short Form, the revised Stress- Related Growth Scale, The Implicit Self-Theories Scale, and the Stress Mindset Scale. Seventy- two parents were randomly assigned to an “intervention group”, and 35 to a comparison group. Members of the intervention group participated in a short synergic growth mindset intervention, created especially for this research. Six months after the intervention, all participants re-completed the same questionnaires. The intervention significantly increased growth mindset and SRG and decreased parental stress. This study demonstrates the effectiveness of a short-term intervention in promoting growth mindsets, reducing parental stress, and fostering SRG among parents of children with a chronic disorder. These findings are particularly important since many parents of children with chronic disorders often exhibit fixed mindset patterns due to their children’s slow progress in various developmental domains.

## Introduction

The parenting experience of children with autism spectrum disorder (ASD) is often perceived as stressful because of the distinctive challenges and uncertainties involved (Hayes & Watson, 2013). In the present study, we tested whether a short-term intervention would be effective in promoting a growth mindset, reducing parental stress, and promoting stress-related growth (SRG) among Arab parents of children with ASD. This neurodevelopmental disorder is predominantly diagnosed during early childhood. It is characterized by substantial impairments in reciprocal social communication and social interaction, along with restricted, repetitive patterns of behavior or interests (Ilias et al., 2018; American Psychiatric Association [APA], 2013). Parenting a child with autism spectrum disorder (ASD) presents unique challenges that often result in heightened stress levels compared to parenting typically developing children. ASD, typically diagnosed in early childhood, is characterized by significant impairments in social communication and interaction, along with restricted, repetitive patterns of behavior or interests (American Psychiatric Association, 2013; Ilias et al., 2018).

The prevalence of symptoms related to post-traumatic stress disorder (PTSD) among parents of children with autism creates daily challenges and uncertainties associated with raising a child with ASD and can evoke stressful responses (Hayes & Watson, 2013). While these parents show higher levels of PTSD symptoms compared to parents of children with typical development, fewer than 20% reached the clinical threshold for PTSD (Stewart et al., 2020).

The pervasive maladaptive and aggressive behaviors displayed by some children with ASD have been associated with heightened parenting stress and psychopathology (Davis & Carter, 2008), Additionally, the continuous need for specialized therapies, the financial burden of autism-related treatments, and the pervasive social stigma all exacerbate the traumatic aspects of parenting a child with ASD (Montes & Halterman, 2007).

The main factors affecting the level of stress among parents of children with ASD include the child’s unpredictable behaviors, impairments in social communication, restricted and repetitive behaviors (Ilias et al., 2018), negative marital relationships, limited opportunities for social integration within the family, lack of time and mental resources for the parents personal, occupational, and economic development (Johnson et al., 2011). Those parents frequently harbor negative perceptions regarding their children’s advancement across various developmental domains and doubt their ability to contribute to their improvement (Hayes & Watson, 2013). They also tend to view themselves as less competent in their parental role than parents of children with typical development (Giallo et al., 2011). Many parents lower their expectations and become uncertain about the child’s future and family (Wayment et al., 2019). These challenges can be particularly noticeable in Arab societies, where there is a strong stigma against individuals with atypical mental and physical development (Duvdevany & Abboud, 2003).

However, research has shown that facing such significant challenges can sometimes lead to unexpected positive psychological changes. Tedeschi and Calhoun (2004) conceptualized Stress-Related Growth (SRG) as an umbrella term encompassing post-traumatic growth (PTG), which occurs when individuals successfully navigate challenging events. These transformative experiences can lead to enhanced self-perception, deeper interpersonal relationships, altered perspective of life, spiritual and existential growth, and a greater appreciation of life (Tedeschi & Calhoun, 1996, 2004).

This phenomenon represents positive psychological changes such as resilience, enhanced relationships, and spirituality in response to adversity (Tedeschi & Calhoun, 1996). Ord et al. (2020) proposed that SRG manifests primarily through enhancing social and personal resources and developing adaptive coping skills.

Parents of children with ASD have demonstrated SRG through increased personal strength, empowerment, and understanding of their children (Waizbard-Bartov et al., 2019). A recent study examining maternal caregivers of children with ASD (Kim et al., 2022) revealed three key aspects: personal growth, strengthened relationships, and improved coping strategies. These mothers also formed a unique peer support group facilitating their growth.

Research shows that parents of children diagnosed with ASD who experience enhanced SRG often have close family bonds, social support, and a heightened appreciation of life and are more likely to rebound from adversity and adapt positively (Markoulakis et al., 2012).

Supporting the potential for actively developing SRG, findings of a recent study on non-clinical university students who received a ten-session SRG intervention showed significant improvements in SRG scores, with effects persisting during follow-up. In contrast, the control group showed no changes, suggesting their benefit in preventive mental health approaches (Yazıcı-Kabaday & Özteme, 2024).

Individual mindsets are crucial in fostering such growth and reducing stress (Crum et al., 2013; Yeager & Dweck, 2012). Mindset encompasses individual basic beliefs regarding the malleability of human traits and characteristics, as well as stress. Research on mindset distinguishes between two main types: A fixed mindset, characterized by perceiving one’s abilities and personal characteristics as congenital and fixed, and a growth mindset, characterized by perceiving one’s abilities and personal characteristics as flexible, incremental and capable of development through effort, effective strategies, and support from others (Dweck, 2006; Yeager et al., 2019).

Similarly, individuals’ mindsets regarding stress refer to the “stress-can-be-enhancing” and “stress-as-debilitating” mindsets (Crum et al., 2013, 2017). This distinction is based on the understanding that psychophysiological stress responses (e.g., racing heart, increased breathing, and feelings of anxiety) can either hinder or mobilize. The positive, mobilizing functionality is correlated with the delivery of oxygenated blood to the brain and body tissues, which can be directed toward promoting stress as an enhancer rather than debilitating (Crum et al., 2013, 2017).

The mindset of parents of children with ASD plays a crucial role in shaping the development of their children and the well-being of both the parents and their children. Such parents often view themselves as less competent in their role compared to parents of children with typical development (Giallo et al., 2011). A fixed mindset often entails believing that intelligence and abilities are inherent and unchangeable (Dweck, 2006). In contrast, by adopting a growth mindset, parents can embrace the belief that their child has the potential to learn and grow despite the challenges they may face. In the context of parenting a child with ASD, a fixed mindset might result in frustration and a sense of hopelessness. In contrast, a growth mindset might encourage the understanding that abilities can be developed even in individuals with autism.

### Short-Term Mindset Interventions for Changing Mindsets, Reducing Parental Stress, and Promoting SRG

There is growing evidence demonstrating that mindset interventions are effective in reducing stress (Hahm, 2016; Yeager & Dweck, 2012) and promote SRG. Ability-directed interventions focus on enhancing growth mindsets by emphasizing that investing effort and seeking help can improve an individual’s abilities. In these interventions, participants learn about brain plasticity and how abilities can develop over time by embracing challenges, employing effective strategies, and seeking guidance from others (Dweck & Yeager, 2019; Yeager et al., 2019).

Similarly, the objective of the stress mindset intervention (e.g., Crum et al., 2017, 2021; Goyer et al., 2022) is to shift participants’ perspective on stress, from believing it is inherently harmful, to recognize its potential benefits. Participants are educated about the enhancing effects and positive aspects of stress, transforming their mindsets from perceiving “stress-is-debilitating” to seeing it as beneficial to their health, well-being, and performance (“stress-can be-enhancing”). Crum et al. (2013) showed that participants who were taught about the enhancing aspects of stress experienced an increase in positive affect and were more inclined to seek advanced feedback when confronted with a stressful experience or task. In contrast, participants taught about the debilitating aspects of stress, experienced decreases in positive affect throughout the study. In another study, incoming first-year students who participated in a stress mindset intervention and were exposed to the enhancing aspects of stress reported higher levels of positive affect during the stressful exam weeks compared to other students who participated in a stress management intervention and to the group without any treatment (Goyer et al., 2022).

Research has also shown that stress mindset interventions can significantly impact individuals’ self-reported mental health and work performance (Crum et al., 2013, 2017; Goyer et al., 2022).

To the best of our knowledge, no prior research has been conducted on growth mindset among parents of children with chronic disorders such as ASD. Only a few interventions have been implemented for parents, primarily involving parents of children with typical development (e.g.,Moorman & Pomerantz, 2010; Rowe & Leech, 2019). For instance, a single-session growth mindset intervention for parents effectively enhanced the early gesture and vocabulary development of their ten-month-old infants (Rowe & Leech, 2019). In another study, second-grade children showed improved reading and writing skills after their parents participated in a growth mindset intervention. This effect was notably stronger among parents who initially did not possess a growth mindset (Andersen & Nielsen, 2016).

A synergistic intervention targeting both growth and stress-can-be-enhancing mindsets among secondary and post-secondary students was recently applied successfully in a large-scale study on adolescents with typical development (Yaeger et al., 2022). The results of six studies showed that the synergistic mindsets intervention improved stress-related cognitions, cardiovascular reactivity, daily cortisol levels, psychological well-being, academic success, and anxiety during the 2020 COVID-19 lockdowns. Moreover, a comparison of the results achieved by the synergistic method with the results of a growth-mindset-only treatment and a stress-mindset-only treatment showed that neither of the single-mindset treatments reliably reduced measures of stress.

Our main objective was to investigate whether a short-term intervention among parents of children diagnosed with ASD, which is a chronic disorder characterized by enduring stress, would be effective in promoting a parental growth mindset, reducing parental stress, and promoting parental SRG.

To enhance the effect of the short-term intervention, we implemented a synergic intervention, which combined an intervention for promoting a growth mindset regarding the belief in the development of abilities (Dweck, 2006) and a mindset regarding “stress as an enhancer.” This synergic intervention seemed particularly appropriate for Arab parents of children with ASD, given the conservative norms of Arabic societies and their negative stereotypes toward atypical development.

## Methods

### Community Involvement Statement

We established a community advisory board consisting of five parents of children diagnosed with ASD from the Arab community in Israel. This approach aligns with the principles of community-based participatory research (CBPR), fostering trust and engagement between researchers and the community. The advisory board played a key role in refining our questionnaires to ensure that these questionnaires are culturally appropriate and clear to the participants. Additionally, their input on the intervention videos was instrumental in refining the content and presentation to better resonate with the community. This collaboration strengthened the cultural relevance, quality, and overall impact of the research while also highlighting the importance of community involvement in ASD research, particularly in diverse populations.

#### Participants

One hundred and seven Arab married parents, from those 37 couples (mothers and fathers) and 33 mothers without their partners participated in the study, totaling 70 mothers and 37 fathers of 3–6.9-year-old boys (*Mean* = 5.01; SD 1.11) diagnosed with ASD participated in this study. The time elapsed since the child received the ASD diagnosis ranged from 7 months to 5.33 years (*Mean* = 2.44; SD 1.26). According to the Childhood Autism Rating Scale—Second Edition—Standard Version (CARS2-ST; Schopler et al., 2010), which was used by us as an additional tool to confirm the diagnosis, 43 (61.4%) of the children had mild-to-moderate ASD, while 27 (38.6%) had severe ASD.

The number of children in the investigated families from 1 to 6 (*Mean* = 2.87; *SD 1.24).* Only boys were included to minimize within-group variability and because the ratio of ASD in the population is 4.3 boys to one girl (CDC, 2020). The socio-economic status of the families (*Mean* = 3.62; SD 0.95). Among the families, 32.9% (23) live in cities and 67.1% (47) live in villages. Fifty-nine families were Muslim (84.3%), six were Christian (8.6%), and five were Druze (7.1%), which is about their ratios in the Arab community in Israel.

#### Procedure

Parents were recruited through advertisements on social networks, forums for parents of children with ASD, contacts with social workers in social service departments, paramedical staff members in child development centers, managers of communication kindergartens, and associations for parents of children with ASD. This recruitment followed ethical approval from the ethics committee of the faculty of education at the University. The researcher contacted families that met the research criteria and provided them with written information about the study, which included a detailed explanation of the research and the researcher’s contact information. Each participant was contacted individually, given further explanations about the nature of the research, and was asked to sign a consent form for participation.

We included parents of male children aged 3 to 7 years with an established ASD all children were diagnosed by a multidisciplinary team consisting of a neuro-developmental pediatrician/psychiatrist and a developmental psychologist, ensuring a comprehensive evaluation. The children had to reside at home, without any known medical problems, and attended a communication, regular, or integrated kindergarten. Additionally, the children had to have been diagnosed with ASD for at least six months to minimize the influence of the parents’ strong emotional reactions, which usually characterize the first period after receiving the diagnosis (Altiere & Von Kluge, 2009).

Participants were randomly assigned to two groups using a randomized control trial method. We implemented a before-after design. Two-thirds of the parents participated in a short-term mindset intervention (intervention group), and one-third served as a comparison group. Before the mindset intervention, all participants completed several questionnaires. After completing the questionnaires, participants in the intervention group then viewed two videos of about 20 min each regarding “abilities mindset intervention” (Yeager et al., 2019) and stress as enhancing (Crum et al., 2013, 2017).

The questionnaires were administered 6–8 weeks prior to the intervention. Following the intervention, all questionnaires were administered again six months later. Additionally, immediately after completing the second assessment of the questionnaires, the intervention was conducted with the comparison group, ensuring they also had the opportunity to benefit from the intervention.

All the research measures were translated using the Brislin (1970) method of translation and back translation to ensure accuracy and cultural appropriateness. The first author translated all questionnaires into Arabic and back-translated them with an additional research assistant. Both are bilingual Arabic-English speakers and licensed clinicians specializing in working with children with ASD.

#### Measures

##### Demographic Questionnaire

This questionnaire probed main background characteristics, including the parents’ education, the level of social support they received, and their health condition. Additionally, parents were asked to report significant positive and negative life events that occurred in the six months between the intervention and the repeated assessment.

##### The Childhood Autism Rating Scale – Second Edition – Standard Version (CARS-ST; Schopler et al., 2010)

The CARS consists of 15 domains assessing behaviors associated with autism, such as adaptation to change, listening response, verbal communication, and emotional response. Each domain is scored on a 1–4 scale, with higher scores associated with a higher level of impairment. The CARS has good internal consistency (α = 0.91—0.94) (Schopler et al., 1988; Tachimori et al., 2003); interclass correlation for the total score of 0.83—0.94 (DiLalla & Rogers, 1994), and test–retest stability over 1 year of 0.88 for the total score (Schopler et al., 1988).

##### Parenting Stress Index-Short Form (PSI-SF; Abidin, 1995)

The PSI-SF (Abidin, 1995) is a 36 self-report items of parenting stress, with three subscales with 12 items in each subscale: 1. The Parental Distress subscale assesses the distress a parent feels due to personal factors related to parenting, such as lack of social support or parental depression. 2. The Parental-Child Dysfunctional Interaction subscale assesses whether the parent perceives interactions with their child as positive (i.e., rewarding) or negative (i.e., unsatisfying). 3. The Difficult Child subscale assesses the behavioral characteristics of the child that make him or her easy or difficult to manage. The total PSI-SF score is an indicator of the parent’s overall experience of parenting stress. Parents rate each item on a 5-point scale ranging from strongly disagree (1) to strongly agree (5). The PSI-SF is valid and reliable with internal reliability coefficients of α = 0.80–0.87 (Abidin, 1995). Its score is associated with levels of mental distress (Haskett et al., 2006), and it discriminates between parents of children with typical development and parents of children diagnosed with ASD (Brobst et al., 2009).

##### Revised Version of the Stress-Related Growth Scale (SRGS; Boals & Schuler, 2018)

The SRGS scale consists of 15 statements relating to the change that occurred after the stressful events, rated on a 7-point Likert scale ranging from −3 (a very negative change) to 0 (no change) to 3 (a very positive change). It examines the degree of personal growth after stressful events, by measuring changes in five aspects of SRG: the meaning of life, life satisfaction, religiosity, gratitude, and positive interpersonal relationships. The internal reliability is α = 0.93 (Boals & Schuler, 2018), and it is associated with the meaning of life, satisfaction with life, religious commitment, gratitude, and positive relationships with others. It is negatively correlated with mental health (Boals & Schuler, 2018).

##### Implicit Self-Theories Scale (Robins & Pals, 2002)

This questionnaire consists of a five-item scale examining the nature of the parents’ mindsets (fixed versus growth) regarding their child’s abilities. Participants indicate their agreement with each item on a scale of 1 (not very true of my child) to 5 (very true of my child). A high score indicates a fixed mindset toward the child’s abilities. The scale has high reliability (range 0.87–0.90). It also predicts the orientation toward achievement and respondents’ attribution style (Robins & Pals, 2002).

##### Stress Mindset Scale (Crum et al., 2013)

The questionnaire assesses general and specific stress mindsets on a 5-point Likert scale. Respondents indicate their level of agreement (0 “strongly disagree” to 4 “strongly agree) regarding their beliefs about general stress (SMM-G, eight phrases) and regarding their beliefs about specific stress related to the ASD diagnosis (SMM-S, eight phrases). This scale has been validated using several samples, including college students (Crum et al., 2017), and employees at a large financial firm (Crum et al., 2013). The reported reliability of the scales is α = 0.86 for SMM-G and α = 0.80 for SMM-S.

### The Intervention Group

We implemented a two-session growth mindset intervention, each lasting approximately 20 min. The first session included a video that focused on “abilities mindset intervention”, which was grounded in scientific knowledge about the plasticity of the human brain, including children with ASD (Dawson, 2008). The video underscored the idea that embracing challenges, maintaining determination, using effective strategies, and seeking guidance and help, all improve the parents’ abilities and, consequently, the abilities of their child. Additionally, the intervention targeted fixed-trait attributions, which ascribe failures to low inherent ability and foster performance avoidance (Dweck & Yeager, 2019; Yeager et al., 2019). In addition to the scientific knowledge provided and to make the concept more relatable, participants watched personal stories portrayed by actors demonstrating experiences of Arab parents of children with ASD who perceived challenges as opportunities for growth. These parents also shared how adopting a growth mindset towards themselves and their children positively impacted their children’s abilities and development. After watching the first video, the participants’ parents were asked to answer two questions: “Think for a moment, how can you promote your child’s abilities? “and “What would you write/say to another parent dealing with raising a child with ASD, that could strengthen their belief and ability to bring about change in their child?”. The latter questions are meant to promote parents’ internalization of the intervention content by giving advice to imagined others in similar conditions to their own and supporting them. A similar task was performed in previous programs to enhance the intervention’s impact (Crum et al., 2017; Dobronyi et al., 2019; Schleider et al., 2019). This might also play an important role in promoting the intervention’s impact.

The second video addressed the perception of stress as enhancing. It presented scientific information explaining that stress hormones protect the body from harm and aid in cell rebuilding, protein synthesis, and immunity enhancement (Crum et al., 2017). The video covered various stress-related topics, including health and vitality, learning and growth, performance, and productivity. Stress was portrayed as a catalyst for learning, creativity, gaining new perspectives, improving relationships, and leading to SRG (Tedeschi et al., 2017, 2018).

Enhancing messages were incorporated, emphasizing that the stress response improves brain processing, focus, decision-making, memory, immunity, and overall performance. The video also included personal stories presented by actors, describing parents who underwent personal growth experiences following their child’s diagnosis with ASD. Also, the participant’s parents were prompted to reflect on moments of personal growth in their own lives.

### The Comparison Group

In our study, the comparison group was randomly selected from the overall sample. During both time points of the questionnaires, this group did not undergo the intervention, nor did they receive any other interventions from the researchers. The comparison group maintained their usual routine and standard care practices throughout the study. After completing the second assessment, they received the same intervention to ensure equal access to potential benefits.

## Results

### Descriptive Statistics

First, we examined the association between the background and research variables. Separate multivariate analyses of variance (MANOVA) for mothers and fathers did not yield significant differences. The adjusted correlation following Bonferroni correction demonstrated that among mothers, poorer health status, lower social support, and higher autism severity were associated with higher levels of parental stress (correlation coefficients of 0.34, − 0.50, 0.51, respectively, with adjustments, p < 0.004). Autism severity was also linked to a higher fixed mindset (adjusted correlation coefficient of p < 0.004, − 0.47). Among fathers, higher autism severity was associated with higher levels of parental stress (adjusted correlation coefficient of 0.51, p < 0.004). Therefore, background variables, health status, and autism severity served as control variables in all analyses. For mothers, social support was also a control variable.

Our analysis did not reveal significant differences between the background variables of the intervention group and the comparison group. Mann–Whitney tests were conducted No differences were found in the number of positive and negative events experienced by participants in both groups. Table 1 presents the background data and socio-demographic characteristics for mothers and fathers.

**Table 1:** Background data and socio-demographic characteristics for mothers and fathers

Variable	Mothers (n = 70)				Fathers (n = 37)			
	*N (%)*	*M*	*SD*	*Range*	*N (%)*	*M*	*SD*	*Range*
Participant age (years)		32.84	5.56	21–48		38.14	5.19	26–50
Education								
Up to middle school	7 (10%)				7 (18.9%)			
High school	28 (40%)				12 (32.4%)			
Post-secondary (no degree)	12 (17.1%)				11 (29.7%)			
Academic	23 (32.9%)				7 (18.9%)			
Employment								
Salaried	28 (40.0%)				23 (62.2%)			
Self-employed	6 (8.6%)				10 (27.0%)			
Homemaker	34 (48.6%)				–			
Unemployed	2 (2.9%)				4 (10.8%)			
Weekly working hours		26.56	10.70	4–45		43.28	9.70	12–60
Economic status		3.67	0.93	1–5		3.59	1.00	1–5
Health status		4.47	0.76	2–5		4.08	1.06	1–5
Social support		4.85*	1.70	1–7		4.48**	1.78	1–7
Religiosity								
Secular	6 (8.6%)				3 (8.1%)			
Traditional	42 (60.0%)				23 (62.2%)			
Religious	22 (31.4%)				11 (29.7%)			

^*^Cronbach’s α = 0.71, **Cronbach’s α = 0.76

### Correlations Between Growth Mindsets, and Parental Stress and SRG

Table 2 depicts the Pearson correlation between growth mindsets (toward abilities and stress) with parental stress and SRG. The table shows that both growth mindsets correlated negatively with the PS and positively with the SRG.

**Table 2:** Pearson correlations between growth mindsets with parental stress and SRG

SRG	Parental stress	Variable
0.32**	− 0.56***	Growth mindset toward abilities
0.43***	− 0.46***	Growth mindset toward stress
0.46**	− 0.36*	General growth mindset (average of abilities and stress)

*p < .05, **p < .01, ***p < .001

The parents’ mindsets toward abilities and stress were subjected to a two-way analysis of variance with repeated measures. The independent variables were the time of measurements (before/after six months of the intervention)—a within-subject variable, and the “group” (intervention/comparison group)—a between-subjects variable.

The analysis yielded a significant effect for time, and group type (*F*_(2,104)_ = 8.93, *p* < 0.001; Wilk’s *Λ* = 0.85, *η*^*2*^ = 0.15; and (*F*_(2,104)_ = 8.14, *p* < 0.01; Wilk’s *Λ* = 0.87, *η*^*2*^ = 0.14), respectively. The interaction between the measurement time and “group type” was also significant (*F*_(2,104)_ = 15.12, *p* < 0.001; *Wilk’s Λ* = 0.78, *η*^*2*^ = 0.23). To uncover the source of the interaction, separate analyses of variance on the measurement time were conducted in each group. The results yielded a significant effect for the intervention group (*F*_(2,70)_ = 28.18, *p* < 0.001; Wilk’s *Λ* = 0.55, *η*^*2*^ = 0.45), but not for the comparison group (*F*_(2,33)_ = 0.70, *p* = 0.50; Wilk’s *Λ* = 0.96, *η*^*2*^ = 0.04).

**Fig. 1:**
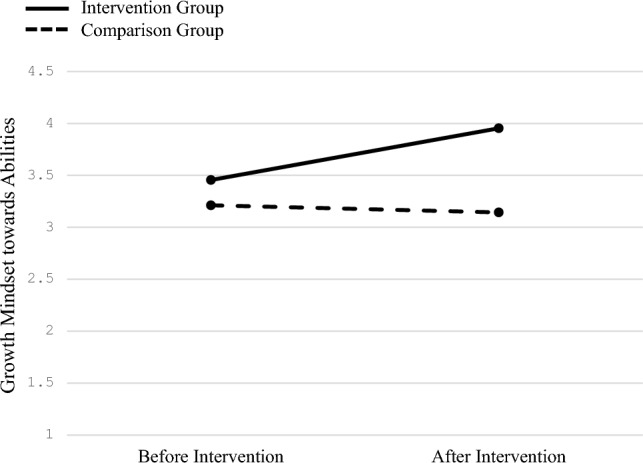
Mean of growth mindset towards abilities by the group type before and after the intervention

**Fig. 2:**
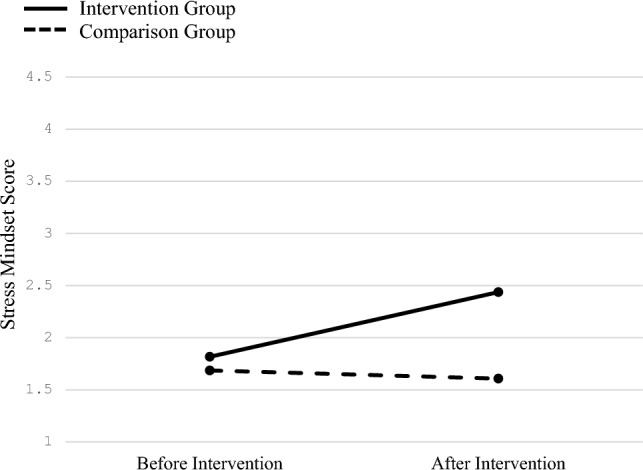
Mean of stress mindset by group type before and after the intervention

**Fig. 3:**
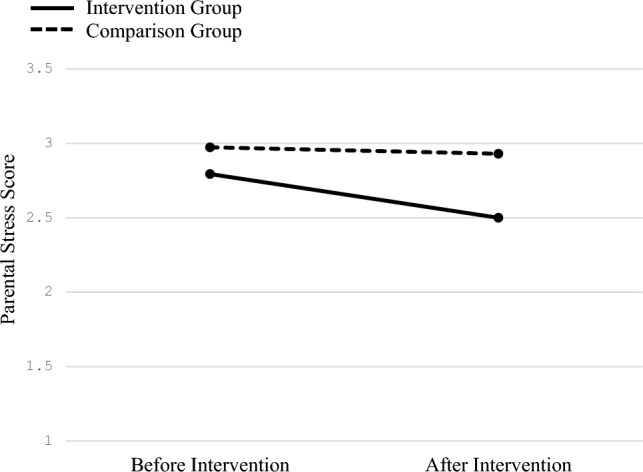
Mean of parental stress by group type before and after the intervention

**Fig. 4:**
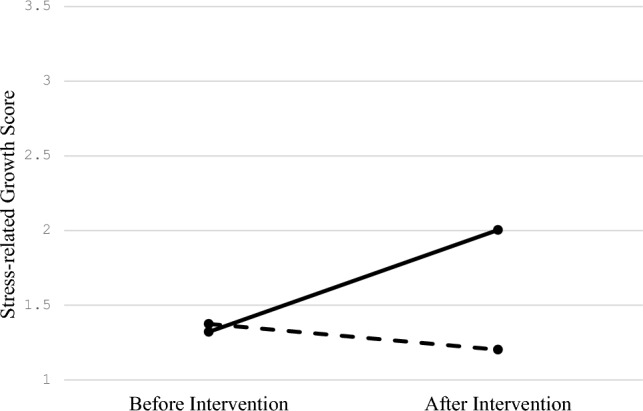
Mean of stress-related growth by group type before and after the intervention

Parental stress level and SRG were subjected to two variance analyses with repeated measures. The independent variables were the time of measurement (a within-subject factor), and the group type (a between-subjects factor). The analyses yielded main effects for the time of measurement on the parental stress and their SRG (*F*_(1,105)_ = 14.37, *p* < 0.001, *η*^*2*^ = 0.12), and *F*_(1,105)_ = 7.37, *p* < 0.01, *η*^*2*^ = 0.07), respectively. Also, a significant interaction effect was found between the type of group and the measurement time on parental stress (*F*(1,105) = 7.90, *p* < 0.01, η2 = 0.07), indicating that the stress level was lower in the second assessment only in the intervention group (Table 3) (Figs. 1, 2, 3 and 4).

**Table 3:** Mindsets toward abilities and stress, parental stress and SRG by time of measurement and the group type

Variable	Intervention group (n = 72)		Comparison group (n = 35)			
	*M*	*SD*	*F*	*M*	*SD*	*F*
Growth mindsets toward abilities						
Before the intervention	3.45	0.85	37.11***	3.21	0.75	0.60
After the intervention	3.95	0.79		3.14	0.89	
Stress mindsets						
Before the intervention	1.82	0.73	45.11***	1.69	0.77	0.36
After the intervention	2.44	0.73		1.61	.86	
Parental Stress						
Before the intervention	2.79	0.54	29.96***	2.97	0.70	0.47
After the intervention	2.50	0.53		2.93	0.67	
SRG						
Before the intervention	1.32	0.77	72.71***	1.37	0.74	4.77
After the intervention	2.00	0.67		1.20	0.85	

***p < .001

In addition, a significant main effect was found for the time of measurement on SRG (*F*(1,105) = 16.02, *p* < 0.001, η^2^ = 0.13) and the type of group (*F*(1,105) = 7.10, *p* < 0.01, η^2^ = 0.10). Moreover, a significant interaction effect was found between the time of measurement and the type of group (*F*(1,105) = 44.99, *p* < 0.001, η^2^ = 0.30). On average, the parents in the intervention group (but not in the comparison group) reported higher levels of SRG in the second assessment.

To better understand the effect of the intervention and determine whether this effect could be explained by changes in the mediating variables (mindset toward abilities and stress), two mediation models were conducted: one related to parental stress and the other to SRG as dependent variables, with the type of group (intervention/control) serving as the independent variable. The analyses were performed using PROCESS version 4.0 by Andrew F. Hayes. As shown in Table 4, the change in mindset toward abilities and stress due to exposure to the intervention mediates the reduction in parental stress but does not promote SRG.

**Table 4:** Indirect paths (intermediaries) in mediation models

Dependent variable	Mediating variable	ab: Beta	95% CI Low	95% CI High
Change in parental stress	Change in mindsets toward abilities	− 0.13	− 0.25	− 0.04
	Change in mindsets toward stress	− 0.11	− 0.23	− 0.01
Change in SRG	Change in mindset toward abilities	0.08	− 0.03	0.24
	Change in mindset toward stress	0.13	− 0.001	0.28

## Discussion

The present research demonstrated that a short-term, synergistic mindset intervention enhanced growth mindsets, reduced parental stress, and promoted SRG among parents of children with ASD. In this regard, the study showcased the effectiveness of mindset interventions for parents of children with a chronic disorder who often view their child’s condition as stressful (Hayes & Watson, 2013). The intervention group displayed significant positive changes in parental mindsets, stress levels, and SRG, while the comparison group showed no changes. All intervention and comparison group participants received ongoing para-medical and psycho-social treatments within their educational environments. The significant changes noted in the intervention group suggest that the video-based mindset intervention provided additional benefits.

The strength of the intervention is remarkable, given the fact that parents of children with ASD typically experience high levels of stress (Hayes & Watson, 2013; May et al., 2015). Remarkably, this effect persisted for at least six months Similar results have been reported in other studies employing brief mindset interventions with other at-risk populations, such as individuals with special educational needs and mental disabilities (e.g., de Carvalho & Skipper, 2020; Verberg et al., 2022), and those with psychopathological conditions, such as anxiety and depressive disorders (e.g., Schleider & Weisz, 2016; Schleider et al., 2019). These results align with meta-analytic findings in various populations, including those with mental health challenges and special needs (Yeager & Dweck, 2012).

The intervention’s efficacy stemmed from integrating two complementary growth mindsets: a growth mindset regarding abilities and a stress-as-enhancing mindset, as documented by Yeager et al. (2022). While Yeager et al. (2022) noted that synergistic mindset interventions may not alter appraisals of uncontrollable stressors, our intervention focused on promoting growth within existing circumstances rather than attempting to control ASD itself. The intervention specifically targeted parents’ perceptions of managing challenges and facilitating growth under these conditions.

Several features contributed to the intervention’s strength. One is the inclusion of audio recordings featuring parents who had faced similar challenges. This element likely promoted participant growth by legitimizing the sharing of feelings and experiences. This aspect is particularly significant for the Arab participants in the present study, who belong to a society that holds negative attitudes toward diversity in general and toward individuals with neurodevelopmental disorders in particular (Khanh et al., 2023). Another feature that probably enhanced the intervention’s strength was encouraging participants to offer advice to other parents in similar circumstances. The practice of providing advice contributed to personal growth through supportive interactions (McKnight & Kashdan, 2009).

Notably, our statistical analysis revealed that the positive change in mindsets toward abilities and stress mediated the reduction in parental stress but did not mediate SRG promotion. It is possible that the intervention itself prompted the parents to appreciate the benefits of their children’s challenging difficulties. The comparably strong effect of the intervention on SRG might suggest that the growth mindset’s mediating effect on SRG was overshadowed by the intervention’s direct effect. This, however, requires further investigation.

Our findings revealed that there were no differences between mothers and fathers in stress levels. The high paternal stress levels observed can be attributed to three key factors. The first is the financial burden; about half of mothers are homemakers, and fathers typically shoulder the primary financial responsibility. Second, Arab society is shifting from traditional to egalitarian parenting roles, creating tension for fathers who must balance traditional expectations with new parenting demands (Haj-Yahia & Lavee, 2018). Third, it might be related to self-selection bias in the sample, as only a portion of fathers participated, suggesting that the participants may represent more involved fathers than the general population.

For mothers, there was a correlation between poorer health status, limited social support, the severity of their children’s autism, and elevated stress levels. The cycle of physical exhaustion, emotional strain, and caregiving demands tends to accumulate, making it especially challenging for mothers to manage the stresses associated with raising a child with severe ASD symptoms (Wah et al., 2024). In addition, research showed that lower social support strongly correlates with increased stress among mothers. Social support is a crucial protective factor, mediating the relationship between stress and life satisfaction for mothers (Khusaifan & El Keshky, 2021). Conversely, our results show that the only stressor found for fathers is the severity of the child’s ASD symptoms, which aligns with other previously reported studies (Soltanifar et al., 2015). Our findings revealed gender differences in stress patterns and growth experiences. While fathers’ stress was primarily linked to their children’s ASD symptom severity (Soltanifar et al., 2015), mothers showed more complex patterns of stress and growth. These gender differences in SRG align with prior research suggesting that women are generally more emotionally expressive and more likely to seek social support—two factors that facilitate SRG (Tedeschi et al., 2017). Fathers, who often prioritize professional responsibilities over caregiving, may have fewer opportunities for parenting-related personal growth. This pattern reflects traditional gender roles in caregiving and suggests the need for targeted interventions that consider these distinct experiences.

Clinical implications derived from these findings indicate that it is essential to develop culturally sensitive interventions tailored to each parent’s unique stressors and growth pathways. Such interventions should consider the broader social and cultural contexts that shape parental roles. Enhancing support systems, particularly for mothers, and creating opportunities for fathers to engage more directly in caregiving may foster both stress reduction and SRG.

This study revealed several methodological limitations. First, the study was conducted only with parents of male children to minimize within-group variability, as the ratio of ASD in the population is 4.3 boys to one girl (CDC, 2020). However, this exclusion of females does not fully represent the general population of children with ASD. Future studies should include both male and female children diagnosed with ASD, allowing for a more comprehensive and representative understanding of parental experiences.

Another limitation is the reliance on the CARS as a confirmatory diagnostic tool. Although the children were initially diagnosed by a multidisciplinary team, using only one standardized measure may not fully capture the complexities of ASD diagnosis. Future studies should include the ADOS, parental reports, and historical data to enhance the rigor of participant diagnosis.

Another limitation concerns the dependence on self-report measures, which may have introduced biases through several methodological artifacts. Participants may have reported self-enhancing scores (Podsakoff et al., 2003). Although self-reports of growth may express real change, they could also be due to illusion, cognitive bias, or a combination of both (Sumalla et al., 2009; Taylor & Brown, 1988). According to Maercker and Zoellner (2004), post-traumatic growth has two sides: a constructive side and a side that can be called a “positive illusion,” which may lead to negative consequences if the feelings of growth are not translated into action. To overcome this limitation, future studies could rely on an assessment of observations on parent–child interactions, in addition to reports from other significant individuals, such as educational and therapeutic agents.

Yeager et al. (2022) showed that despite the significant effect of implementing the synergistic method (growth mindset and stress-can-be-enhancing mindset), the growth mindset-only treatment and the stress mindset-only treatment were ineffective. Due to the difficulty in recruiting enough Arab parents of children with ASD, we were unable to conduct the study on each growth mindset separately. Future research should examine the separate effects of each mindset component by including a growth mindset-only treatment and a stress mindset-only treatment with a synergic treatment.

Given the challenges in recruiting Arab minority children in Israel, we designed our study with an uneven distribution: two-thirds of the participants were assigned to the intervention group and one-third to the comparison group. While this approach helped us gather more data about the intervention’s effects, it introduced methodological limitations. Future research would benefit from a larger sample size and equal allocation between intervention and comparison groups to strengthen the validity of findings.

Furthermore, focusing on a small, specific Arab population means these preliminary insights should be interpreted cautiously when considering their generalization to other populations. Future research should utilize more diverse samples and assess intervention efficacy across various cultural groups. Establishing a community advisory board could ensure culturally sensitive adaptations while preserving the intervention’s core advantage of being both brief and cost-effective. This approach aligns with the need to develop sustainable, easily implementable solutions to benefit a wider range of parents seeking support. As demonstrated in recent literature (Khanh et al., 2023), stigma can significantly moderate parental experiences across different cultural settings. Therefore, future studies should explicitly investigate how cultural contexts and stigma interact to influence intervention outcomes, helping to clarify the broader applicability of our findings beyond the specific population examined.

Since our study focused on a small, specific Arab population, it is essential to recognize that these findings cannot be generalized without careful adaptation to other cultural and social contexts. Although similar interventions may effectively reduce stress and promote SRG among parents of children with other chronic disorders, their success would depend on adjustments tailored to the unique needs of diverse populations. Establishing a community advisory board could help ensure these adaptations are pertinent and culturally sensitive. Furthermore, emphasizing a short- and low-cost intervention meets the need to create sustainable solutions that are easy to implement and maintain over time, benefiting a broader range of parents who need support.

As with similar interventions, it is essential to note that this short-term intervention approach did not intend to resolve all the parents’ challenges. Additional follow-up assessments could provide further insights into the long-term impact of the intervention. Unfortunately, such assessments were beyond the scope of the current study. While our study provides promising results, an essential next step should involve assessing the generalizability of the intervention’s effects on parents of children with other chronic disorders and developmental delays.

## Conclusion

This research provides valuable insights into the effectiveness of synergistic growth mindset interventions for parents of children with ASD. The intervention successfully enhanced growth mindsets, reduced parental stress, and promoted SRG. We believe that the durability of the intervention’s effects, along with its low cost per single parent, ease of administration, and short duration, holds the promise that similar interventions, with the necessary adaptations for specific groups, could prove effective in reducing stress and promoting stress-related growth among parents of children with other chronic disorders.

Our findings underscore the need for gender-sensitive interventions that address both shared and unique challenges mothers and fathers face (Benson, 2006; Vacca, 2013). For mothers, strengthening social support networks and improving healthcare access can serve as protective factors against stress (Boyd, 2002). For fathers, programs should emphasize developing practical caregiving strategies while addressing work-family role conflicts. These targeted approaches can enhance overall family resilience and improve outcomes for both parents and their children with ASD. Our approach is consistent with the broader trend in ASD research toward developing more personalized and family-centered support strategies (Karst & Van Hecke, 2012). Future research should focus on developing and evaluating the effectiveness of these interventions, contributing to the growing body of evidence-based practices in autism support.
